# Disorders of Calcium Metabolism: Hypocalcemia and Hypercalcemia

**DOI:** 10.7759/cureus.12420

**Published:** 2021-01-01

**Authors:** Mohammad Tinawi

**Affiliations:** 1 Medicine, Indiana University School of Medicine Northwest-Gary, Gary, USA; 2 Nephrology, Nephrology Specialists, Munster, USA

**Keywords:** hypocalcemia, hypercalcemia, electrolyte disorders, calcium metabolism, calcium sensing receptor

## Abstract

Calcium (Ca^+2^) is a divalent cation that plays a critical role in numerous body functions such as skeletal mineralization, signal transduction, nerve conduction, muscle contraction, and blood coagulation. Ca^+2^ metabolism is linked to magnesium (Mg^+2^) and phosphate metabolism. Ca^+2 ^homeostasis is dependent on intestinal absorption, bone turnover, and renal reabsorption. The hormonal regulators of these processes are the parathyroid hormone (PTH), calcitriol {1,25-dihydroxyvitamin D [1,25(OH)_2_D]}, and serum ionized Ca^+2^. Cloning of the Ca^+2^-sensing receptor (CaSR) has greatly advanced the understanding of Ca^+2^ metabolism. Disorders of Ca^+2^ metabolism are easily recognized because Ca^+2^ is included in routine chemistry panels. Measurement of ionized Ca^+2^ is the preferred way to ascertain the diagnosis of hypocalcemia and hypercalcemia.

## Introduction and background

Forms of serum calcium

**Figure 1:**
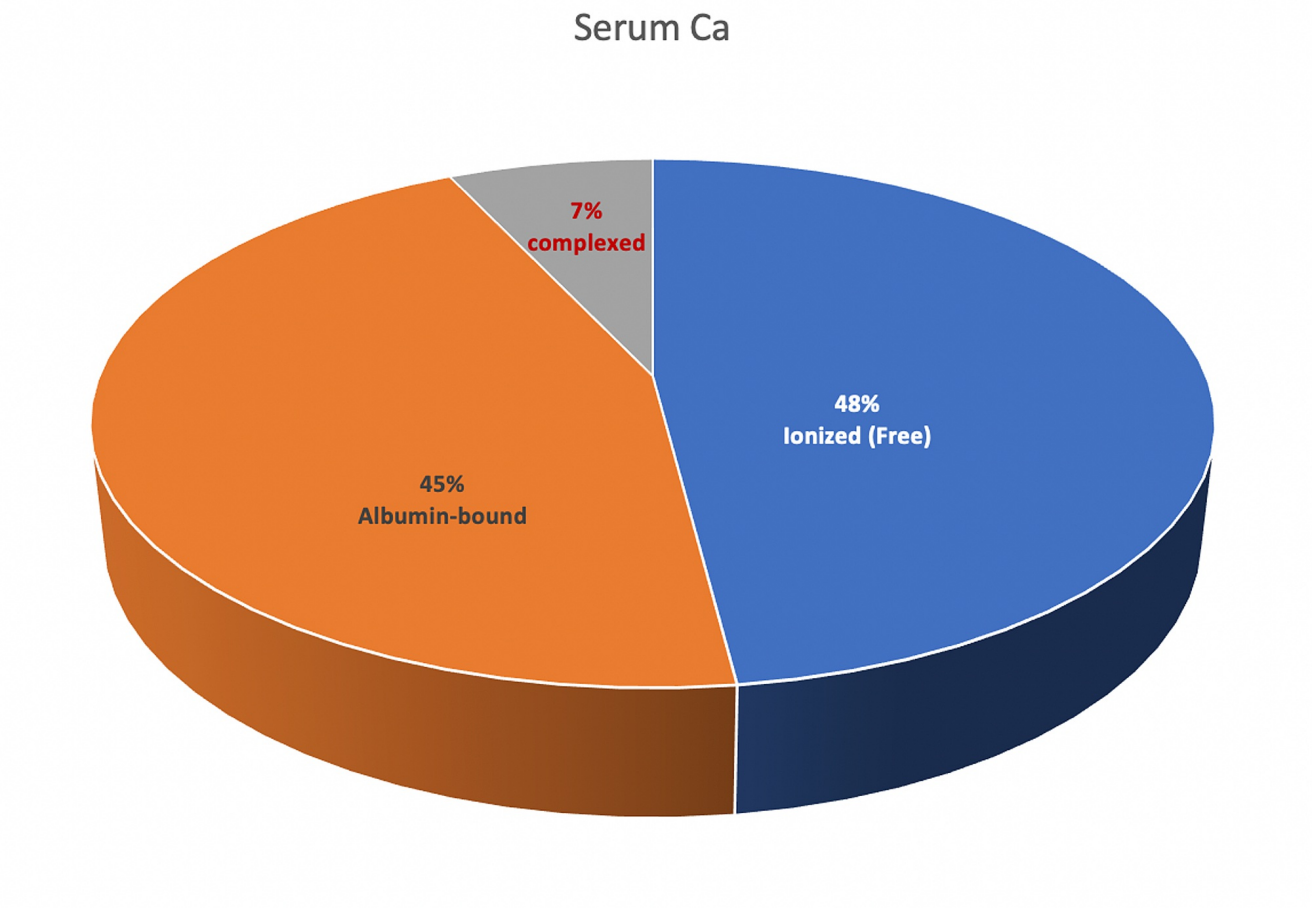
The three different forms of serum calcium Complexed calcium is bound to anions such as citrate, oxalate, carbonate, and phosphate

Normal total serum calcium (Ca^+2^) concentration is 8.8-10.4 mg/dl, and this is equivalent to 4.4-5.2 mEq/l or 2.2-2.6 mmol/l [1]. To convert from mmol/l to mEq/l, multiply by +2, which is the valence of calcium. To convert from mmol/l to mg/dl, multiply by 40 (the atomic weight of Ca^+2^) and divide by 10 (i.e., multiply by 4). The normal value for ionized Ca^+2^ is about half of total serum Ca^+2^, 4.4-5.2 mg/dl, 2.2-2.6 mEq/l, or 1.10-1.30 mmol/l. Serum Ca^+2^ exists in three forms: ionized (free; 48%), protein-bound (mostly to albumin and less to globulins; 45%), and complexed (bound to citrate, oxalate, carbonate, and phosphate; 7%), as shown in Figure 1. Both ionized and complexed Ca^+2^ are diffusible (ultrafilterable by the kidney), while protein-bound Ca^+2^ is not [2].

Intracellular Ca^+2^ is bound to calmodulin and other Ca^+2^-binding proteins. Hypoalbuminemia will lead to hypocalcemia due to a decrease in protein-bound Ca^+2^. To correct for hypoalbuminemia, the following formula is used: 

Corrected total serum Ca^+2^ (mg/dl) = measured serum Ca^+2^ (mg/dl) + 0.8 (4.0 - serum albumin g/dl).

For example, if serum Ca^+2^ is 7.8 mg/dl and serum albumin is 2.5 g/dl, corrected serum Ca^+2^ = 7.8 + 0.8 (4.0 - 2.5) = 9.0 mg/dl; therefore, total serum Ca^+2^ is normal in this case and does not require replacement. Similarly, an increase in albumin by 1.0 g/dl will lead to a 0.8 mg/dl increase in total serum Ca^+2^. This equation is not always accurate, especially in patients with stage 3-5 chronic kidney disease (CKD) [3]. Ionized Ca^+2 ^should be checked whenever feasible to ascertain the diagnosis of hypocalcemia or hypercalcemia.

Calcium distribution in the body

The human body contains about 1,000-1,300 g of Ca^+2^, making Ca^+2^ the fifth most abundant element in the body [1]. About 99.3% of total body Ca^+2^ is in the bone (skeleton) and teeth, 0.6% is in soft tissues, and 0.1% resides in the extracellular fluid (ECF), including 0.03% in plasma [4]. Intracellular Ca^+2^ concentration is very low (about 100 nM), yet it is essential for several critical functions such as signal transduction, nerve conduction, muscle contraction, and blood coagulation. Ca^+2^ in the skeleton is complexed with phosphorus mainly as hydroxyapatite, which gives the bone its mechanical characteristics. It is important to know that only 1% of Ca^+2^ in the bone can immediately equilibrate with extracellular Ca^+2^. 

Incidence and prevalence of calcium metabolism disorders

Hypercalcemia is fairly common with a prevalence of approximately 1-4% in the general population and 0.17-3% in hospitalized populations [4]. Hypocalcemia is significantly more prevalent in hospitalized patients (10-18%). In those hospitalized in the intensive care unit, the prevalence of hypocalcemia can be as high as 70-80% [4]. It is important to also recognize that the prevalence of hypomagnesemia in the intensive care setting is as high as 65%, which contributes to the high prevalence of hypocalcemia in this patient population.

## Review

Calcium homeostasis 

Intestinal Absorption of Calcium

**Figure 2:**
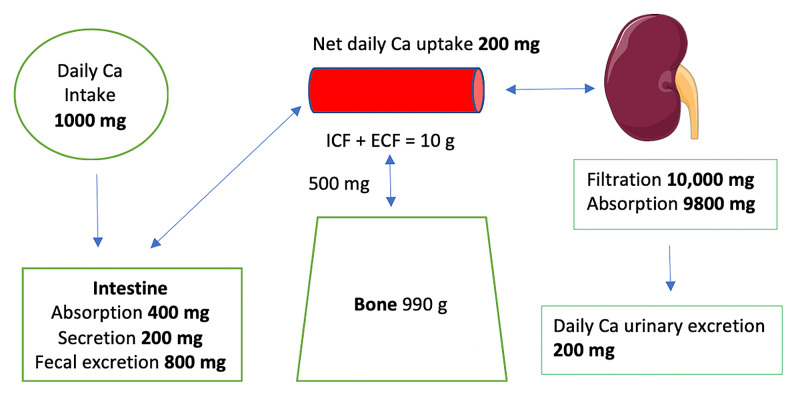
Calcium homeostasis Approximately 500 mg of calcium is exchanged daily between the ECF and the bone ICF: intracellular fluid; ECF: extracellular fluid Image of the kidney is courtesy of Servier Medical Art licensed under a Creative Commons Attribution 3.0 Unported License. https://smart.servier.com

The average daily intake of Ca^+2^ is about 1,000 mg, of which 400 mg is absorbed in the small intestine. About 200 mg is excreted with intestinal secretions. Therefore, net absorption is 200 mg (about 20%); the remaining 800 mg is excreted in the stool [2]. About 500 mg of Ca^+2^ are exchanged daily between the bone and the ECF. Of the 10,000 mg of Ca^+2^ filtered through the kidneys, 9,800 mg (98%) are reabsorbed by the renal tubules, and approximately 200 mg are excreted, which equals the net amount absorbed in the small intestine (Figure 2).

In the small intestine, Ca^+2^ is absorbed both paracellularly (passive absorption through tight junctions) and transcellularly (active absorption). Paracellular absorption dominates when Ca^+2^ intake is high, while transcellular absorption dominates when Ca^+2^ intake is low [1]. Active absorption of Ca^+2^ is under the control of calcitriol [1,25(OH)_2_D]. Transcellular Ca^+2^ absorption occurs via two epithelial Ca^+2 ^channels that belong to the transient receptor potential (TRP) superfamily and specifically to the vanilloid subfamily (TRPV) [5]. These two channels are transient receptor potential vanilloid 5 (TRPV5; pronounced trip V5) and TRPV6. Free Ca^+2^ exits the cell via the sodium-calcium (Na^+^-Ca^+2^) exchanger.

Hormonal Regulation of Calcium Homeostasis

Ca^+2^ homeostasis is dependent on three processes: intestinal absorption, bone turnover (Ca^+2^ exchange with the bone), and renal reabsorption [1]. The hormonal regulators of these processes are the parathyroid hormone (PTH), calcitriol [1,25(OH)_2_D], which is the most active form of vitamin D, and serum ionized Ca^+2^. The receptors for these hormonal regulators are the PTH receptor (PTHR), the vitamin D receptor (VDR), and the calcium-sensing receptor (CaSR) respectively [6].

The Calcium-Sensing Receptor (CaSR)

The CaSR is a G protein-coupled receptor that regulates PTH secretion from the parathyroid glands. The CaSR senses extracellular ionized Ca^+2^. When serum Ca^+2^ is high, the CaSR is activated with a subsequent increase in renal Ca^+2^ excretion (calciuria) and inhibition of PTH secretion [7]. PTH inhibition decreases the release of Ca^+2^ from the bone and inhibits the synthesis of calcitriol. Inhibition of calcitriol synthesis also reduces mobilization of Ca^+2 ^from bone and decreases active intestinal absorption of Ca^+2^. These effects will help in restoring Ca^+2^ towards normal levels [8]. The opposite effect is seen when serum Ca^+2^ is low. The CaSR is inactivated with a subsequent decrease in renal Ca^+2^ excretion and an increase in PTH secretion. PTH stimulation increases the release of Ca^+2^ from the bone and enhances the synthesis of calcitriol. Calcitriol mobilizes Ca^+2^ from the bone and increases active Ca^+2^ absorption in the intestine. These effects will help in restoring Ca^+2^ towards normal levels [9]. The CaSR is also expressed in the basolateral membranes of the thick ascending limb (TAL) of the loop of Henle.

Renal Calcium Handling

**Figure 3:**
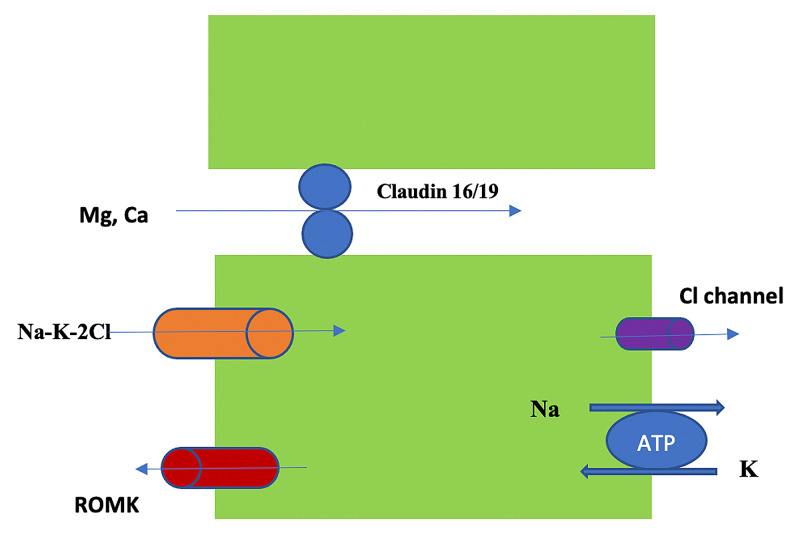
Calcium and magnesium reabsorption in the TAL via the paracellular route The process is passive and depends on sodium and potassium uptake via the Na-K-2Cl pump Na-K-2Cl: sodium-potassium-2 chloride pump; ROMK: the renal outer medullary potassium channel; TIL: thick ascending limb; ATP: adenosine triphosphate Courtesy of Tinawi M, Disorders of Magnesium Metabolism: Hypomagnesemia and Hypermagnesemia. Arch Clin Biomed Res, 4(3): 205-220, 2020; an open-access article distributed under the terms and conditions of the Creative Commons Attribution (CC-BY) license 4.0

In the kidney, the proximal tubule (PT) reabsorbs 60-70% of filtered Ca^+2^, the TAL reabsorbs 20%, the distal convoluted tubule (DCT) reabsorbs 10%, and the collecting duct (CD) reabsorbs 5% [2]. Regulation of Ca^+2^ excretion in the kidney occurs at the terminal nephron. Ca^+2^ reabsorption in the PT is 85% via the paracellular route (passive) [10]. Active transport via the apical membrane (transcellular) is responsible for the remaining 15% and is enhanced by calcitonin and PTH. In the TAL, absorption is both paracellular and transcellular but mostly paracellular [11]. As in the PT, transcellular (active) Ca^+2^ transport in the TAL is enhanced by calcitonin and PTH. Claudin-16 interacts with claudin-19 (both are tight junction proteins) forming a cation-selective tight junction protein complex that enables paracellular Ca^+2^ (and Mg^+2^) transport in the TAL (Figure 3).

Claudin-14 blocks paracellular Ca^+2^ reabsorption in the TAL in response to increased serum Ca^+2^ level [12]. Ca^+2^ reabsorption in the DCT is entirely active via the transcellular route through TRPV5 channels [13]. Hormonal regulations of Ca^+2^ and phosphate are tightly linked, while there is no hormonal system that significantly controls Mg^+2^ metabolism [14]. Phosphate homeostasis is regulated by PTH, calcitriol, fibroblast growth factor 23 (FGF-23), and FGF/Klotho receptor complex [2]. FGF-23 is secreted from the bone in response to an increase in serum phosphate level. It results in phosphaturia and a decrease in calcitriol production with a subsequent decrease in intestinal phosphate (and Ca^+2^) absorption. Increased PTH secretion leads to phosphaturia. While both PTH and FGF-23 are phosphaturic, they have the opposite effect on calcitriol (FGF-23 decreases and PTH increases the renal production of calcitriol).

Parathyroid Hormone (PTH)

PTH is the main regulator of renal Ca^+2 ^reabsorption. A decrease in serum ionized Ca^+2^ (hypocalcemia) inactivates the CaSR in the parathyroid glands and subsequently stimulates PTH secretion. PTH and calcitriol enhance renal Ca^+2 ^reabsorption in the DCT via the transcellular (active) route [13]. Moreover, PTH stimulates bone resorption by the osteoclasts and increases the secretion of calcitriol, which in turn stimulates intestinal Ca^+2^ and phosphate absorption by activating the VDR. Conversely, hypercalcemia decreases PTH secretion by activating the CaSR and the above actions are reversed. The hormonal response keeps serum Ca^+2^ in a narrow physiologic range [1]. Therefore, the function of the CaSR in the parathyroid glands is to change PTH secretion depending on serum ionized Ca^+2^ level. Both Mg^+2^ and Ca^+2^ bind to the CaSR in the parathyroid glands and the kidney; however, each has a distinct binding site. Mg^+2^ plays a role in PTH modulation by acting on the CaSR [15,16]. PTH secretion is stimulated in acute hypomagnesemia and suppressed in hypermagnesemia. It is important to note that profound hypomagnesemia suppresses (rather than stimulates) PTH secretion and increases PTH resistance in the bone leading to hypocalcemia [9,10].

Vitamin D

Calcitriol is the most active form of vitamin D and is produced by tubular renal cells. 25-hydroxyvitamin D [25(OH)D] is produced in the liver and is converted to calcitriol by 1α-hydroxylase [3,8,17]. Calcitriol enhances intestinal Ca^+2^ and phosphate absorption in addition to renal ​​​​​Ca^+2^ reabsorption in the DCT.

Renal Calcium Reabsorption

Volume expansion increases urine Na^+ ^and Cl^-^ excretion and subsequently decreases renal Ca^+2^ absorption and the reverse is true in volume contraction. In metabolic alkalosis, bound hydrogen ions dissociate from albumin, which increases the fraction of albumin available for ionized Ca^+2^ binding [2]. Therefore, metabolic alkalosis leads to hypocalcemia. Acute and chronic metabolic acidosis leads to hypercalcemia because hydrogen is buffered in the bone with subsequent release of Ca^+2^ and calcinuria. Ionized Ca^+2^ changes by 0.12 mg/dl for each 0.1 change in pH. Inhibition of the sodium-potassium chloride cotransporter 2 (NKCC2) in the loop of Henle by loop diuretics enhances Ca^+2^ excretion in the urine [8]. Thiazide diuretics are associated with hypercalcemia and hypocalciuria due to enhanced Ca^+2^ reabsorption in the PT (following Na+ and water reabsorption due to volume contraction) and in the DCT (Table 1).

**Table 1:** Factors affecting renal calcium reabsorption PTH: parathyroid hormone

Factors that increase renal Ca reabsorption	Factors that decrease renal Ca reabsorption
Calcitriol	Calcitriol deficiency
Hyperparathyroidism (increased PTH)	Hypoparathyroidism (decreased PTH)
Hypocalcemia	Hypercalcemia
Metabolic alkalosis	Metabolic acidosis and respiratory acidosis
Thiazide diuretics, amiloride	Loop diuretics, mannitol
Hypovolemia	Hypervolemia

Hypercalcemia increases calcitonin production by the C cells in the thyroid gland. Calcitonin inhibits bone resorption by the osteoclasts and increases renal Ca^+2^ and phosphate excretion [18]. Hypercalcemia activates CaSR in the basolateral membrane of the TAL. CaSR inhibits the renal outer medullary potassium channel (ROMK), which in turn inhibits K^+ ^recycling in the TAL; subsequently, the activity of the NKCC2 is decreased, which lowers the positive transepithelial voltage. The final outcome of this sequence of events is a decrease in paracellular transport of Na^+^, Mg^+2,^ and Ca^+2 ^(increased urinary excretion of Na^+^, Mg^+2^, and Ca^+2^) [2]. This explains why severe hypercalcemia leads to volume depletion and why normal saline (and not loop diuretics that lead to further volume depletion) is the first step in the management of severe hypercalcemia. 

Hypocalcemia

Hypocalcemia is defined as serum Ca^+2 ^of <8.8 mg/dl (2.2 mmol/l or 4.4 mEq/l). Hypocalcemia is easily diagnosed because Ca^+2^ is included in routine chemistry panels. As in hypercalcemia, Ca^+2^ should be corrected in case of hypoalbuminemia or hyperalbuminemia. It is preferable to obtain ionized Ca^+2^ to ascertain the diagnosis, especially in critically ill patients in whom pH variation changes Ca^+2^ binding to albumin [19]. As mentioned above, metabolic alkalosis increases Ca^+2^ binding to albumin and decreases ionized Ca^+2^. Hypocalcemia stimulates PTH release, which increases renal production of calcitriol; both hormones increase serum Ca^+2^ by the mechanism mentioned above.

Etiology

Hypocalcemia is more common than hypercalcemia in hospitalized patients. PTH can be low, normal, or high. Hypocalcemia due to PTH deficiency is associated with low or low normal PTH and hyperphosphatemia, while other causes are associated with high PTH. Vitamin D deficiency, acute pancreatitis, hungry bone syndrome, and Mg^+2^ deficiency cause hypocalcemia with normal or low serum phosphate [8]. The hungry bone syndrome is seen post parathyroidectomy in patients with severe primary hyperparathyroidism. The most common causes of hypocalcemia are listed in Table 2.

**Table 2:** Causes of hypocalcemia PTH: parathyroid hormone; CKD: chronic kidney disease

Causes
PTH deficiency: hereditary (e.g., isolated congenital hypoparathyroidism, DiGeorge syndrome) or acquired hypoparathyroidism, post-parathyroidectomy and post-thyroidectomy, post-parathyroid glands radiation
Vitamin D deficiency or resistance [lack of sun exposure, inadequate dietary intake, intestinal malabsorption (steatorrhea), hepatic disease, CKD, osteomalacia, rickets]
Increased Ca^+2^ uptake as in rhabdomyolysis, tumor lysis syndrome, hungry bone syndrome (post-parathyroidectomy), or acute pancreatitis
Ca^+2^ malabsorption due to small bowel pathologies such as short bowel syndrome and sprue
Acute hyperventilation (which results in respiratory alkalosis)
Low dietary intake of Ca^+2^ (rare in the absence of intestinal malabsorption or vitamin D deficiency)
Osteoclastic bone metastases as in prostate cancer and small cell lung cancer
Pseudohypoparathyroidism, which is a rare genetic disorder characterized by resistance to PTH actions
Hypermagnesemia and profound hypomagnesemia (due to suppression of PTH secretion)
Acute severe hyperphosphatemia
Intake of Ca^+2^-binding ingredients such as oxalate, phosphate, and cellulose
Medications: bisphosphonate, denosumab, cinacalcet, etelcalcetide
Massive blood transfusion due to binding of ionized Ca^+2^ with citrate
Critical illness
Pseudohypocalcemia caused by some gadolinium contrast agents (e.g., gadoversetamide) due to interference with total calcium laboratory assay

Primary hypoparathyroidism can be due to antibodies against the parathyroid glands or activating antibodies against the CaSR. In either case, hypocalcemia ensues. Transient or permanent hypoparathyroidism can occur post thyroidectomy [20]. Activating mutations of the CaSR result in hereditary hypoparathyroidism, which is characterized by marked hypercalciuria [21]. Many patients with advanced CKD have secondary hyperparathyroidism (high PTH) and unlike primary hyperparathyroidism; they have low or low normal Ca^+2^ due to calcitriol deficiency. High doses of vitamin D will cause hypercalcemia in advanced CKD patients. Therefore, in CKD patients, Ca^+2^ can be low, normal, or high, and in many patients with advanced CKD (stages 4 and 5 and patients on dialysis), serum phosphate is high even in the presence of vitamin D deficiency. Both cinacalcet and etelcalcetide are calcimimetics (positive allosteric CaSR modulators) approved for the treatment of secondary hyperparathyroidism in dialysis patients. Both medications can cause hypocalcemia. One study has found hypocalcemia in 55% of patients admitted to the critical care unit of a tertiary care center [22]. Critical illness hypocalcemia is multifactorial and is attributed to vitamin D deficiency, abnormal PTH secretion and action, circulating catecholamines, medication adverse effects, and citrated blood transfusion. 

Manifestations

Most patients with chronic hypocalcemia are asymptomatic. The clinical manifestations of hypocalcemia are a function of its severity and rapidity of onset. Hypocalcemia can manifest with muscle weakness, fatigue, confusion, depression, and memory loss [8]. Severe manifestations are seen in acute hypocalcemia and include seizures, tetany, paresthesias, laryngospasm, anxiety, and QT interval prolongation. Trousseau’s sign (carpopedal spasm) is carpal spasm during blood pressure measurement when the cuff is kept inflated over the systolic blood pressure for three minutes, resulting in forearm ischemia [23]. Chvostek’s sign is facial muscle twitching when the facial nerve is tapped near the jaw angle about 2 cm anterior to the earlobe. Both signs are due to neuromuscular excitability. Chronic hypocalcemia, as in hypoparathyroidism, can be associated with dry keratotic skin, ridged nails, and course brittle hair [24].

Evaluation

In addition to serum Ca^+2^, albumin, and ionized Ca^+2^, other electrolytes, especially phosphate and Mg^+2^, are measured. ECG is needed in severe hypocalcemia. Further evaluation includes measurement of urea, creatinine, PTH, 25(OH)D, 1,25(OH)_2_D, and 24-hour urinary Ca^+2 ^and phosphate. Patients with elevated PTH and creatinine due to CKD can have hypocalcemia associated with secondary hyperparathyroidism. Low PTH points toward hypoparathyroidism. If PTH is elevated (the expected response to hypocalcemia), and 25(OH)D is low, the patient has vitamin D deficiency. Patients with vitamin D-dependent and vitamin D-resistant rickets have elevated PTH and normal 25(OH)D levels [4].

Management

Symptomatic patients (usually Ca^+2^ of <7.6 mg/dl or 1.9 mmol/l; ionized Ca^+2^ of <1 mmol/l) are treated with intravenous Ca gluconate (93 mg or 2.32 mmol elemental Ca/1g). Ca chloride (273 mg or 6.80 mmol elemental Ca/1g) can be given if central venous access is available. Cardiac monitoring is recommended during intravenous Ca^+2^ replacement, especially in patients on digoxin [25]. Asymptomatic patients are treated with oral Ca^+2^ supplements, usually Ca carbonate or Ca citrate; 1 g of Ca carbonate contains 400 mg of elemental Ca (40%), while 1 g of Ca citrate contains 211 mg of elemental Ca (21%). Ca acetate is used as a phosphate binder in patients with CKD. Vitamin D should be replaced if deficient [23]. Either vitamin D2 (ergocalciferol) or D3 (cholecalciferol) can be given. Oral calcitriol at a dose of 0.25-1 mcg/day is particularly helpful. Hypomagnesemia should be corrected. Hyperphosphatemia in patients with hypoparathyroidism is managed with a low phosphate diet and phosphate binders. In patients with hypoparathyroidism, Ca^+2^ should be kept in the low normal range because overtreatment with Ca^+2^ and vitamin D supplementations will lead to hypercalciuria, nephrolithiasis, nephrocalcinosis, and soft-tissue calcifications [23]. Recombinant human parathyroid hormone (rhPTH 1-84, Natpara) is approved in the US for the management of hypocalcemia in hypoparathyroidism in addition to Ca^+2^ and vitamin D [26]. However, its distribution is restricted. It has a black box warning due to the potential risk of osteosarcoma. Thiazide diuretics lower urinary Ca^+2^ excretion and may be helpful in patients with hypercalciuria.

Hypercalcemia

Hypercalcemia is defined as serum Ca^+2^ of level >10.4 mg/dl (2.6 mmol/l or 5.2 mEq/l). Hypercalcemia can be mild (Ca^+2^ of 10.5-11.9 mg/dl), moderate (Ca^+2^ of 12-13.9 mg/dl), or severe (hypercalcemic crisis; Ca^+2^ of ≥14 mg/dl) [18]. Patients with mild hypercalcemia are often asymptomatic.

Manifestations

Symptoms are non-specific and may overlap with other electrolyte disorders. As in hypocalcemia, the clinical manifestations of hypercalcemia are a function of its severity and rapidity of onset. The symptoms include fatigue, weakness, anxiety, and increased sleepiness. This is followed by nausea, vomiting, abdominal pain, and constipation. Renal manifestations include polyuria, kidney stones, and nephrocalcinosis. Other manifestations include bone pain, headache, hypertension, shortened QT interval, and rarely, stupor and coma [18]. Severe acute hypercalcemia can result in acute kidney injury (AKI) (due to severe dehydration), nephrogenic diabetes insipidus (NDI), and cardiac arrhythmias [4]. Someone has proposed the mnemonic “stones, bones, abdominal moans and psychic groans” to remember the manifestations of hypercalcemia.

Etiology

Hypercalcemia is most commonly caused by enhanced bone resorption; it can also result from enhanced intestinal absorption or decreased renal Ca^+2^ excretion (Table 3).

**Table 3:** Causes of hypercalcemia PTH: parathyroid hormone; CKD: chronic kidney disease; PTHrP: parathyroid hormone-related protein; CaSR: Ca^+2^-sensing receptor

Causes
PTH excess (primary hyperparathyroidism) due to parathyroid gland adenoma (80% of cases) or hyperplasia (10-15% of cases). Primary hyperparathyroidism is part of the multiple endocrine neoplasia (MEN) 1 and 2A. Parathyroid carcinoma is rare
Humoral hypercalcemia of malignancy
Osteolytic bone metastases as in multiple myeloma, and metastatic breast and lung cancers
Milk-alkali syndrome (increased intestinal absorption of Ca^+2^ due to excessive intake of Ca^+2^, antacids, and vitamin D)
25(OH)D toxicity (usually due to excess intake of over-the-counter supplements)
1,25(OH)_2_D excess as in excessive intake, lymphoma, and granulomatous disorders such as sarcoidosis, tuberculosis, leprosy, berylliosis, histoplasmosis, and Farmer’s lung
Immobilization
Paget’s disease
Thyrotoxicosis, acromegaly, pheochromocytoma, acute adrenal insufficiency
Thiazide diuretics (hypercalcemia is usually mild, and hyperparathyroidism should be excluded), lithium, theophylline, growth hormone, recombinant human PTH (teriparatide), and hyperalimentation solutions
Adynamic bone disease (decreased bone formation) as in patients with end-stage renal disease (ESRD) due to the inability of the bone to take up Ca^+2^
Excess intake of dietary Ca^+2^ in patients with CKD and in children
Vitamin A toxicity (hypervitaminosis A)
Neonatal severe hyperparathyroidism (homozygous CaSR-inactivating mutations)
Familial hypocalciuric hypercalcemia (FHH), which is due to heterozygous CaSR-inactivating mutations
Hypercalcemia of pregnancy (uncommon) due to the production of PTHrP

Most patients with hypercalcemia have either primary hyperparathyroidism or malignancy. Primary hyperparathyroidism predominates in ambulatory patients with hypercalcemia, while malignancies predominate in hospitalized patients. Hypercalcemia in the course of a malignancy carries a poor prognosis [27]. Hypercalcemia can be the first clue to the presence of an occult malignancy. The incidence of hypercalcemia in cancer patients is as high as 30% [28]. Primary hyperparathyroidism and malignancy are responsible for 80-90% of hypercalcemia cases [28]. Malignancy-associated hypercalcemia can be severe and occasionally life-threatening [29]. It is either humoral or due to osteolytic bone metastases. Humoral hypercalcemia of malignancy is responsible for 80% of hypercalcemia due to malignancy. It is mediated by parathyroid hormone-related protein (PTHrP), which enhances osteoclastic activity [30]. Examples include renal cell carcinoma, adenocarcinoma of the ovary and breast, and squamous cell carcinoma of the lung, esophagus, and cervix [31]. PTHrP has the same effect as PTH on target cells and both hormones have a common receptor [8]. Inactivating mutations in the CaSR are the cause of neonatal severe hyperparathyroidism in the case of homozygous mutations, and familial hypocalciuric hypercalcemia (FHH) in the case of heterozygous mutations [7]. Inactivating mutations of the CaSR mimic the effects of PTH. FHH is an autosomal dominant disorder associated with moderate hypercalcemia. FHH patients also have normal or moderately high PTH, hypophosphatemia, and hypermagnesemia. FHH does not result in severe symptomatic hypercalcemia. Milk-alkali syndrome (calcium-alkali syndrome) is caused by increased intestinal absorption of Ca^+2^ due to high intake of Ca^+2^ and vitamin D, especially when taken with antacids (alkali) [32,33]. It is associated with the kidneys' inability to excrete excess Ca^+2^. Patients can also have nephrocalcinosis [8]. The name of the syndrome comes from the practice of ingesting a large amount of milk that is high in Ca^+2^ for the treatment of peptic ulcer disease in the past when effective medications were unavailable. In some patients, the source of Ca^+2^ may not be immediately evident; for example, each piece of nicotine-substitute gum contains 94 mg of elemental Ca^+2^.

Diagnosis

Hypercalcemia is diagnosed when total serum Ca^+2^ is >10.4 mg/dl and ionized Ca^+2^ is >5.3 mg/dl [4]. Hypercalcemia workup includes a detailed history and physical examination with emphasis on medications and supplementations including vitamin D and Ca^+2^. Hypercalcemia in primary hyperparathyroidism is chronic and usually mild and most patients are asymptomatic. Mild to moderate hypophosphatemia can be seen. Cervical ultrasound and 99mTc-sestamibi scintigraphy may help in the localization of parathyroid adenomas. A variant of primary hyperparathyroidism is named normocalcemic primary hyperparathyroidism due to normal serum Ca^+2^ and elevated PTH level [34]. The diagnosis of hypercalcemia is easily made because Ca^+2^ is included in routine chemistry panels. Measurement of other electrolytes such as Na^+^, K^+^, Mg^+2^, and phosphate in addition to renal function tests is needed. Serum Ca^+2^ should be corrected for albumin in cases of hypoalbuminemia or hyperalbuminemia. Measurement of ionized Ca^+2^ is preferable. A study by Obi et al. has reported that the majority of hemodialysis patients with high ionized Ca (hypercalcemia) will be misdiagnosed as normocalcemic if total serum Ca^+2^ (whether corrected for albumin or not) is measured [35]. The same authors have devised the following formula for calculating corrected total Ca^+2^ in hemodialysis patients [36]:

Corrected total serum Ca^+2^ (mg/dL) = 1.35 x total serum Ca^+2^ (mg/dL) - 0.65 x serum albumin (g/dL) - 0.15 x serum phosphorus (mg/dL) + 0.3.

Of note, the authors reported that patients with hidden hypercalcemia had a higher mortality rate when compared to patients with normal serum Ca^+2^ (ionized Ca^+2 ^of 1.16-1.32 mmol/l), [adjusted hazard ratio of approximately 1.75 (95% confidence interval: 1.11-2.75)].

Once the diagnosis is ascertained, PTH should be measured. PTH is appropriately suppressed in all of the above conditions except primary hyperparathyroidism. It can be elevated in FHH as well. The further evaluation depends on suspected etiology. Measurement of 24-hour urine Ca^+2^ is critical in patients suspected of having primary hyperparathyroidism or FHH. If a 24-hour urine collection is not feasible, a random urine Ca^+2^/creatinine ratio can be obtained. In hypercalciuria, the ratio is >0.03. Measurement of PTHrP is done in patients with known or suspected malignancy. Elevated alkaline phosphatase is seen in bone lysis. 1,25(OH)_2_D level is measured in patients suspected of having hypercalcemia due to sarcoidosis or lymphoma. 25(OH)D is measured if patient history raises the possibility of vitamin D toxicity. It is critical to distinguish primary hyperparathyroidism from FHH. In primary hyperparathyroidism, urinary Ca^+2^ is high (>200 mg in 24-hour urine or urine Ca^+2^/creatinine ratio of >0.03), while in FHH it is inappropriately normal or low (urine Ca^+2^/creatinine ratio of <0.02) [4,7]. Genetic testing and detailed family history are needed to ascertain the diagnosis of FHH.

Management

The most critical step in the management of severe hypercalcemia is volume repletion with 0.9% isotonic saline [37]. The value of loop diuretics in the management of severe hypercalcemia is questionable. Loop diuretics should never be given prior to volume repletion [38]. They may have a role in patients who develop hypervolemia [31]. Bisphosphonates (particularly intravenous zoledronate and pamidronate) are indicated for the treatment of malignancy-associated hypercalcemia [39]. This class of drugs inhibits bone resorption and 1,25(OH)_2_D synthesis. A complete response takes two to four days; hence, repeat administration over a short interval will lead to hypocalcemia. Bisphosphonates should be used with caution in patients with CKD. The dose should be lowered, and the infusion rate should be slowed [40]. For example, if a patient has CKD stage 4, zoledronate can be given at a dose of 2 mg (rather than the standard 4 mg dose) over two hours (rather than the standard 15 minutes). Denosumab is a fully human monoclonal antibody that targets the receptor activator of NF-κB ligand (RANKL). It is an effective option for refractory hypercalcemia [41]. Denosumab can result in severe hypocalcemia especially in patients with advanced CKD. Calcitonin-Salmon is expensive and of limited value in the management of severe hypercalcemia [31]. It is given subcutaneously (SQ) or intramuscularly (IM). Tachyphylaxis develops quickly. Corticosteroids are particularly effective in hypercalcemia due to granulomatous diseases such as sarcoidosis because they inhibit the abnormal production of calcitriol [42]. Recalcitrant hypercalcemia, especially in patients with acute kidney injury or advanced CKD, will respond to hemodialysis utilizing a low Ca^+2^ dialysate bath of ≤2 mEq/l [43]. 

Once acute and severe hypercalcemia is treated, the underlying cause should be addressed. Patients with symptomatic primary hyperparathyroidism (nephrolithiasis, moderate or severe hypercalcemia, osteoporosis, fragility fractures, or hypercalciuria) are treated surgically with parathyroidectomy unless contraindicated [44]. Guidelines have been published regarding the management of asymptomatic primary hyperparathyroidism [45]. Cinacalcet (a calcimimetic drug that increases the sensitivity of CaSR to extracellular Ca^+2^) is routinely used in the chronic management of secondary hyperparathyroidism in patients on renal replacement therapy. It can also be used in patients with severe hypercalcemia due to primary hyperparathyroidism [46]. A five-year study in patients with mild to moderate primary hyperparathyroidism showed that cinacalcet reduced PTH level and normalized Ca^+2^ with no change in z-scores of areal bone mineral density (aBMD) [47]. Cinacalcet was well-tolerated by the patients.

## Conclusions

There are three Ca^+2^-regulating hormonal systems: PTH, vitamin D, and calcitonin. CaSR plays a critical role in Ca^+2^ homeostasis. Ca^+2^ level is maintained by the interplay between the above hormones and the bowel (Ca^+2^ absorption), kidneys (Ca^+2^ reabsorption and excretion), and bone (Ca^+2^ uptake and release). The most common causes of hypocalcemia are PTH and vitamin D deficiencies, while the most common causes of hypercalcemia are primary hyperparathyroidism and malignancies. Hypercalcemia is frequently encountered in malignancies and carries a poor prognosis. Aggressive hydration and bisphosphonates are the basis of treatment. Hypocalcemia is managed with the replacement of Ca^+2^, vitamin D, and Mg (if indicated). However, excessive replacement of Ca^+2^ and vitamin D should be avoided.
